# Metabolic Analyses of Nitrogen Fixation in the Soybean Microsymbiont Sinorhizobium fredii Using Constraint-Based Modeling

**DOI:** 10.1128/mSystems.00516-19

**Published:** 2020-02-18

**Authors:** Carolina A. Contador, Siu-Kit Lo, Siu H. J. Chan, Hon-Ming Lam

**Affiliations:** a School of Life Sciences and Center for Soybean Research of the State Key Laboratory of Agrobiotechnology, The Chinese University of Hong Kong, Shatin, Hong Kong SAR, China; b Department of Chemical and Biological Engineering, Colorado State University, Fort Collins, Colorado, USA; Lawrence Berkeley National Laboratory

**Keywords:** bacteroid, metabolic model, nitrogen fixation, rhizobia, symbiosis

## Abstract

Rhizobia are soil bacteria able to establish symbiosis with diverse host plants. Specifically, Sinorhizobium fredii is a soil bacterium that forms nitrogen-fixing root nodules in diverse legumes, including soybean. The strain *S. fredii* CCBAU45436 is a dominant sublineage of *S. fredii* that nodulates soybeans in alkaline-saline soils in the Huang-Huai-Hai Plain region of China. Here, we present a manually curated metabolic model of the symbiotic form of Sinorhizobium fredii CCBAU45436. A symbiosis reaction was defined to describe the specific soybean-microsymbiont association. The performance and quality of the reconstruction had a 70% score when assessed using a standardized genome-scale metabolic model test suite. The model was used to evaluate *in silico* single-gene knockouts to determine the genes controlling the nitrogen fixation process. One hundred forty-one of 541 genes (26%) were found to influence the symbiotic process, wherein 121 genes were predicted as essential and 20 others as having a partial effect. Transcriptomic profiles of CCBAU45436 were used to evaluate the nitrogen fixation capacity in cultivated versus in wild soybean inoculated with the microsymbiont. The model quantified the nitrogen fixation activities of the strain in these two hosts and predicted a higher nitrogen fixation capacity in cultivated soybean. Our results are consistent with published data demonstrating larger amounts of ureides and total nitrogen in cultivated soybean than in wild soybean. This work presents the first metabolic network reconstruction of *S. fredii* as an example of a useful tool for exploring the potential benefits of microsymbionts to sustainable agriculture and the ecosystem.

**IMPORTANCE** Nitrogen is the most limiting macronutrient for plant growth, and rhizobia are important bacteria for agriculture because they can fix atmospheric nitrogen and make it available to legumes through the establishment of a symbiotic relationship with their host plants. In this work, we studied the nitrogen fixation process in the microsymbiont Sinorhizobium fredii at the genome level. A metabolic model was built using genome annotation and literature to reconstruct the symbiotic form of *S. fredii*. Genes controlling the nitrogen fixation process were identified by simulating gene knockouts. Additionally, the nitrogen-fixing capacities of *S. fredii* CCBAU45436 in symbiosis with cultivated and wild soybeans were evaluated. The predictions suggested an outperformance of *S. fredii* with cultivated soybean, consistent with published experimental evidence. The reconstruction presented here will help to understand and improve nitrogen fixation capabilities of *S. fredii* and will be beneficial for agriculture by reducing the reliance on fertilizer applications.

## Abstract (precis)

Nitrogen is the most limiting macronutrient for plant growth, and rhizobia are important bacteria for agriculture because they can fix atmospheric nitrogen and make it available to legumes through the establishment of a symbiotic relationship with their host plants. In this work, we studied the nitrogen fixation process in the microsymbiont Sinorhizobium fredii at the genome level. A metabolic model was built using genome annotation and literature to reconstruct the symbiotic form of *S. fredii*. Genes controlling the nitrogen fixation process were identified by simulating gene knockouts. Additionally, the nitrogen-fixing capacities of *S. fredii* CCBAU45436 in symbiosis with cultivated and wild soybeans were evaluated. The predictions suggested an outperformance of *S. fredii* with cultivated soybean, consistent with published experimental evidence. The reconstruction presented here will help to understand and improve nitrogen fixation capabilities of *S. fredii* and will be beneficial for agriculture by reducing the reliance on fertilizer applications.

## INTRODUCTION

Rhizobia are diazotrophic Gram-negative bacteria able to infect and form nitrogen-fixing nodules on compatible host plants (1). These soil bacteria are distributed across six genera: *Rhizobium*, *Sinorhizobium*/*Ensifer*, *Bradyrhizobium*, *Allorhizobium*, *Azorhizobium*, and *Mesorhizobium* (2). Symbiotic nitrogen-fixing bacteria provide a major source of nitrogen to plants, especially legumes. Most of the species within the family Leguminosae (Fabaceae) can establish a symbiotic association with rhizobia to obtain biological nitrogen (3–5). The interactions between the host plant and rhizobia occur in three stages: recognition and production of specific signal molecules by rhizobia (6), formation of root nodules and newly differentiated tissue in the roots of host plants (7, 8), and differentiation of rhizobia into their symbiotic form, bacteroids, which are able to reduce atmospheric nitrogen to ammonia inside the nodules (5, 9, 10). The symbiotic association between legume and rhizobium involves the release of ammonia to the plant cell cytosol by the nitrogen-fixing bacteroid in exchange for organic acids provided by the host plant. This symbiotic relationship contributes to nitrogen enrichment of the soil, improves plant growth, and reduces the need for chemical fertilizers (11). These features highlight the huge potential of exploiting this interaction for sustainable agriculture. However, the details and degree of the symbiosis are very strain specific and host specific (12), and deeper understanding of the interactions at the metabolic level is required to optimize it.

Among rhizobia, Sinorhizobium fredii is able to form nitrogen-fixing nodules with important legume food crops such as soybeans (Glycine max and Glycine soja), pigeon pea (Cajanus cajan), and cowpea (Vigna unguiculata) (13). In particular, *S. fredii* is a fast-growing soybean rhizobium and dominates in alkaline-saline soil in the Huang-Huai-Hai Plain region in China (14, 15). The *S. fredii* strain CCBAU45436 represents one of the dominant sublineages of *S. fredii* that nodulate soybeans in northern China (16). Recently, the complete genome sequence for CCBAU45436 was fully assembled (17). The CCBAU45436 multipartite genome consists of a chromosome (cSF45436, 4.16 Mb), a chromid (pSF45436b, 1.96 Mb), a symbiotic plasmid (pSF45436a, 0.42 Mb), and two accessory plasmids (pSF45436d, 0.20 Mb, and pSF45436e, 0.17 Mb). Evidence suggests a distinct role for each of these forms of DNA in rhizobial adaptations to symbiotic conditions (17–19). CCBAU45436 is able to induce effective nodules on the roots of the wild soybean accession W05 (*Glycine soja* W05) and the cultivated soybean accession C08 (Glycine max C08). Symbiotic nitrogen fixation by rhizobia in legume root nodules involves several simultaneous biological processes, including biological nitrogen fixation carried out by bacteroids, carbon-nitrogen metabolism, and exchange of nutrients and cofactors between the host plant and bacteroid (20). A better understanding of the nitrogen fixation process is required to facilitate the optimization of symbiotic nitrogen fixation to further increase legume productivity. The differential nitrogen-fixing capacity of the representative *S. fredii* strain, CCBAU45436, in symbiosis with Glycine max C08 versus with *Glycine soja* W05 (21) provides a unique opportunity to analyze the host-specific nitrogen-fixing metabolism of *S. fredii*. The constraint-based modeling approach has been successfully used to integrate genomic and high-throughput metabolomic data to systematically analyze the metabolic capabilities of other rhizobial species to get new insights into the complex process of symbiotic nitrogen fixation carried out in mature nodules (19, 22–25).

Here, we present a metabolic network reconstruction, *i*CC541, for the symbiotic form of the soybean microsymbiont, *S. fredii* CCBAU45436. This is the first manually curated reconstruction of the *S. fredii* bacteroid. The performance and quality of the reconstruction were examined using a standardized genome-scale metabolic model test suite (26). Consistency between model predictions and available experimental observations was evaluated. Flux balance analysis (27) was used to analyze the physiological capabilities of the bacterium under different symbiotic nitrogen fixation conditions. Symbiotic genes were identified and compared with experimental evidence. Finally, the transcriptome profiles of CCBAU45436 at the symbiotic stage within the nodules of cultivated (C08) and wild (W05) soybeans were used to evaluate the nitrogen fixation capacity of *S. fredii* bacteroids.

## RESULTS

### Formulation of metabolic network reconstruction of the symbiotic form of *S. fredii* CCBAU45436.

**FIG 1:**
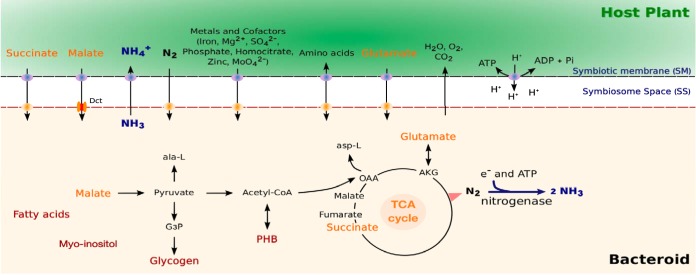
Schematic representation of main metabolic pathways and transport systems in soybean microsymbiont *S. fredii* CCBAU45436. Carbon sources in the plant cytosol are transported across the symbiosome and bacteroid membranes in exchange for fixed nitrogen. Malate and succinate enter the TCA cycle to be metabolized. Transport of cofactors required for nitrogen fixation is indicated. Dct, C_4_-dicarboxylate transport system; ala-L, alanine; asp-L, aspartate; OAA, oxaloacetate; AKG, 2-oxoglutarate; G3P, glyceraldehyde-3-phosphate; PHB, poly-3-hydroxybutyrate.

A metabolic reconstruction (*i*CC541) was developed to represent the symbiotic form of the microsymbiont *S. fredii* CCBAU45436. Metabolic reconstructions previously generated (19, 24) were used as a starting point, with 303 reactions selected in total from both models. Reactions used as the template are indicated in Table S1 in the supplemental material. The draft model was manually curated using information from literature, the *S. fredii* strain pathway database in Kyoto Encyclopedia of Genes and Genomes (KEGG) (28), automated reconstruction databases (29), and other available databases to ensure that it captures the biochemical and physiological knowledge available on nitrogen fixation. A schematic representation of the nutrient and cofactor exchanges between the host plant and the bacteroid is shown in Fig. 1.

10.1128/mSystems.00516-19.2 TABLE S1 List of reactions included in the reconstruction model with their description, associated genes, and their reference numbers in various databases. Download Table S1, XLSX file, 0.1 MB. Copyright © 2020 Contador et al. 2020 Contador et al. This content is distributed under the terms of the Creative Commons Attribution 4.0 International license.

A symbiosis reaction was defined to integrate the essential and characteristic elements of the soybean-rhizobia symbiosis association. Specifically, the legume-rhizobium symbiosis determines the accumulation of metabolites inside the microsymbionts related to carbon and energy storage. *S. fredii* is able to nodulate different legumes and form determinate or indeterminate nodules depending on the host species (30). In particular, poly-3-hydroxybutyrate (PHB), glycogen, and lipids were included in the symbiosis reaction, as *S. fredii* has been shown to accumulate these compounds in determinate soybean nodules (31–34). The synthesis of storage compounds is important for managing the carbon source and reducing power supplied by the host plant. However, the role of carbon storage compounds in the symbiotic process differs depending on the rhizobium strain (12, 20). Furthermore, bacteroid development and nitrogen fixation are dependent on the amino acids supplied by the host plant (35, 36). Therefore, the uptake and export of various amino acids such as glutamate, alanine, aspartate, and arginine between the bacteroid and host plant were also considered in the symbiosis reaction. Bacteroids isolated from soybean nodules possess an active uptake system for glutamate supplied by the plant cytosol (37, 38). Experimental evidence shows that alanine and aspartate can be supplied to the plant by the bacteroid when malate is provided as a carbon source (39). Meanwhile, arginine also plays a role in the symbiotic process, since the decarboxylation products of arginine are the precursors of polyamines (40, 41). Legume root nodules can contain high levels of polyamines, and it plays a role in the regulation of nutrient exchange (42, 43). Transcriptome data have shown that genes associated with polyamine biosynthesis are upregulated in the symbiosis stage (44). We have also included valine and isoleucine in modeling the symbiotic capacity of *S. fredii*, because their biosynthetic pathways have been indicated as essential for effective symbiosis in *Sinorhizobium* and *Rhizobium* (45), and two transposon Tn*5*-induced isoleucine and valine auxotrophs of *S. fredii* HH303 were unable to form effective nodules on soybean as well (46). Moreover, nitrogenous base production has also been demonstrated to be essential for the formation of effective soybean nodules, and *S. fredii* purine and pyrimidine mutants form ineffective nodules in their hosts (42, 43, 46–49). Therefore, purine and pyrimidine metabolism also constitute a component of the reconstruction. However, symbiosis-defective mutants were not genetically characterized, and further experiments are required to explain this phenotype.

Additionally, symbiotic cofactors are key for the activity of nitrogenase and were therefore added to the symbiosis reaction. Nitrogenase is the enzyme responsible for the reduction of atmospheric nitrogen supplied by the host plant to ammonia. The nitrogenase complex consists of two enzymes: dinitrogenase reductase and dinitrogenase (5, 10). Dinitrogenase reductase is a dimeric iron (Fe) protein and dinitrogenase is a tetrameric iron-molybdenum (FeMo) protein. Nitrogenase activities depend on the availability of energy (ATP), low-potential electrons (iron-sulfur clusters), and microaerobic conditions (5, 50). One of these cofactors is homocitrate, which is a component of the iron-molybdenum cofactor of nitrogenase. From the genome sequence of CCBAU45436, no homocitrate synthase has been annotated; thus, the host plant has to provide homocitrate to the bacteroid to synthesize nitrogenase (51). MucR1 has been identified as a transcription regulator associated with the expression of ion transporters linked to phosphate, molybdenum, zinc, iron, and sulfur. Symbiotic defects were observed in *S. fredii mucR1* mutants (52). Additional evidence highlights the role of phosphate in the nitrogen fixation process (33). After nitrogen, phosphate is the most limiting macronutrient, and a high demand for it has been reported in the nodules of nitrogen-fixing legumes (53). Heme and nicotinate are also synthesized by bacteroids, which are supplied to the houseplant for the synthesis of leghemoglobin to maintain the microaerobic condition for nitrogen fixation (36, 46, 47). Other cofactors include pyridoxine (19, 24), magnesium (54), and glutathione (55, 56). The symbiosis equation is a combination of the components described above and it is provided in Table S1. The element balance was checked, and the chemical formulae of the symbiotic product was determined to ensure a molecular weight (MW) of 1 g mmol^−1^ (57).

### Evaluation of *i*CC541 reconstruction.

*i*CC541 describes 538 reactions encoded by 541 genes as well as 508 metabolites. Table 1 summarizes the main properties of *i*CC541. *i*CC541 was evaluated using three methods. First, the reconstruction was validated using experimental data reported in the literature to ensure the model is able to capture unique characteristics of the nitrogen fixation capacity of *S. fredii*. The conditions tested and captured by the model can be found in Table 2. Published experimental data represent the current knowledge status of an organism, and the validation procedure tests the biological accuracy of the reconstruction. Strain-specific metabolic capabilities included carbon sources and phenotypes of mutant strains.

**TABLE 1:** *i*CC541 statistics

Parameter	Value
No. of ORFs^*a*^	541
Chromosome (*n* [%])	441 (82)
Chromid (*n* [%])	81 (15)
Symbiotic plasmid (*n* [%])	11 (2)
Accessory plasmids (*n* [%])	7 (1)
Total reactions (*n*)	535
Metabolic conversions (*n*)	491
Reactions with ORF assignments (*n*)	474
% of reactions with ORF	96
Reactions without ORF (*n*)	17
% of reactions without ORF	4
Transport reactions (*n*)	24
Transport reactions with ORF assignments (*n*)	17
% of transport reactions with ORF	71
Exchange reactions (*n*)	23
No. of metabolites	508

a ORF, open reading frame.

**TABLE 2:** Published experimental data used during the validation process

Phenotype or metabolic characteristics	Reference(s)
Reduction of nitrogen to ammonia by nitrogenase reaction	15
Malate as carbon source	86
Succinate as carbon source	86
Alanine production by bacteroid	64
Production of PHB	51
*pyrC* mutant: Fix^−^	42
*pyrF* mutant: Fix^−^	48
C_4_-dicarboxylate requirement	62
*purL* mutant: Fix^−^	47, 49
*purQ* mutant: Fix^−^	47
*idhA* mutant: decrease nitrogen fixation	66
Glutamate requirement: enhances N_2_ fixation	38
*znuA* mutant: reduce nitrogen fixation	17
*ptsSCAB* mutant: reduce nitrogen fixation	52
*cobO* mutant: reduce number of nodules	43

**FIG 2:**
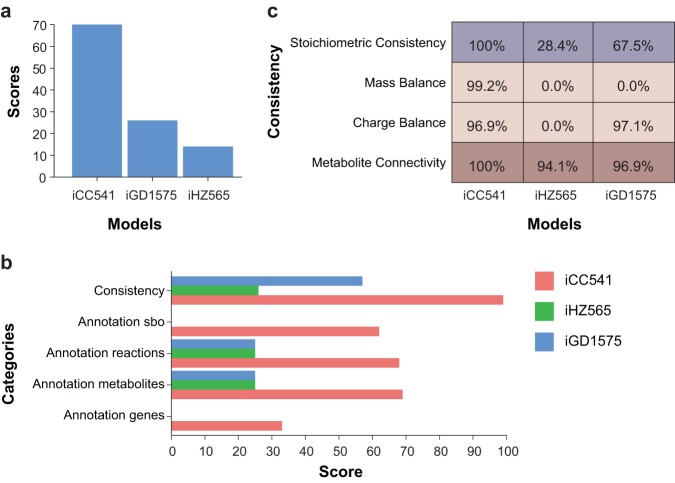
Scores and consistency assigned to *i*CC541, *i*HZ565, and *i*GD1575 by memote. (a) Total scores. (b) Scores by main categories: consistency and annotations (systems biology ontology [sbo], reactions, metabolites, and genes). (c) Consistency across subsections.

Additionally, the *i*CC541 formulation was assessed using a standardized genome-scale metabolic model test suite called memote (26). This metabolic model test approach provides a series of comparisons to benchmark metabolic models and facilitates quality control during model reconstruction. Scores are assigned to properties such as consistency (stoichiometry, mass and charge balance, and metabolite connectivity) and annotation (database cross-references and ontology terms). Scores represent the degree of completeness with respect to the tested property, with a maximum achievable score of 100%. An overall score of 70% was assigned to *i*CC541 by memote (Fig. 2a). Consistency, metabolites, reactions, genes, and systems biology ontology (sbo) annotations achieved scores of 85%, 68%, 69%, 33%, and 62%, respectively (Fig. 2b). The subsections under consistency, namely, stoichiometric consistency, mass balance, charge balance, and metabolite connectivity, were assigned scores of 100%, 99.2%, 96.9%, and 100%, respectively (Fig. 2c). A maximum score in each of these subsections is desirable to ensure reliable model predictions. These scores are comparable to those for well-curated models of Escherichia coli (E. coli core model and *i*AF1260b) and Saccharomyces cerevisiae (*i*MM904) from the Biochemical, Genetic, and Genomic (BiGG) knowledge base (58). *i*CC541 has significantly improved quality in terms of model consistency and annotations compared to that of the published Sinorhizobium meliloti reconstructions, *i*GD1575 and *i*HZ565 (25, 59), which were also tested by memote in this study using the published Systems Biology Markup Language (SBML) files (Fig. 2). The full report returned by memote for *i*CC541 and the two S. meliloti models can be found in Fig. S1.

10.1128/mSystems.00516-19.1 FIG S1 Snapshot of reports obtained from memote for comparison of *i*CC541 with S. meliloti models in HTML format. Model tests are divided in functionality libraries to evaluate core and organism-specific model properties. Core tests are independent of the modeled organism and complexity involved in the model formulation. Each test is divided into scored and unscored sections. The maximum achievable score is 100%. Unscored sections are related to general statistics and specific characteristics of some models such as the quality of the biomass reaction. Download FIG S1, PDF file, 0.5 MB. Copyright © 2020 Contador et al. 2020 Contador et al. This content is distributed under the terms of the Creative Commons Attribution 4.0 International license.

Finally, the simulation performance of *i*CC541 was compared against the S. meliloti models. Inconsistencies reported by memote were solved in order to facilitate cross-comparisons between models. For that purpose, mass and charge imbalances and energy-generating cycles (ATP) (60) were fixed for the S. meliloti models. The symbiosis reactions were scaled to make sure the total mass of the material through the symbiosis reactions was the same in all three models in order to compare symbiosis yields (symbiosis product in grams per millimole carbon) (Table 3). The symbiosis reaction flux was maximized under microaerobic conditions for each model: 0.0107, 0.0145, and 0.0130 g mmol^−1^ were obtained for *i*CC541, *i*HZ565, and *i*GD1575, respectively. Similar yields relative to nitrogen uptake were observed for *i*CC541 and *i*GD1575: 0.0747 and 0.0906 g mmol^−1^ nitrogen, respectively, whereas *i*HZ565 predicts a higher yield with 0.1774 g mmol^−1^. C/N ratios obtained under standard nitrogen fixation conditions (defined according to literature) were comparable in *i*CC541 and *i*GD1575, with *i*HZ565 showing a lower ratio. The number of share reactions, metabolites, and genes between *i*CC541 and the S. meliloti models are indicated in Table 4.

**TABLE 3:** Comparison of symbiosis yields for *S. fredii* (*i*CC541) and S. meliloti (*i*HZ565 and *i*GD1575)

Model	Symbiosis yield (g product/mmol) on:		C/N
	Carbon	Nitrogen	
*i*CC541	0.0107	0.0747	0.1427
*i*HZ565	0.0145	0.1774	0.1431
*i*GD1575	0.0130	0.0906	0.0815

**TABLE 4:** Comparison of *i*CC541 against *i*HZ565 and *i*GD1575

Property	*i*CC541 (*n*)	No. (%)^*a*^	
		Shared by *i*HZ565	Shared by *i*GD1575
Genes	541	407 (75)	400 (74)
Metabolic conversions	491	461 (94)	414 (84)
Metabolic conversions with same directionality	491	455 (93)	291 (59)
Transport reactions	24	16 (67)	16 (67)
Exchange reactions	23	20 (87)	23 (100)
Metabolites	508	488 (96)	434 (85)

a Percentage of coverage in *i*CC541.

### Symbiotic pathway utilization analyses.

**FIG 3:**
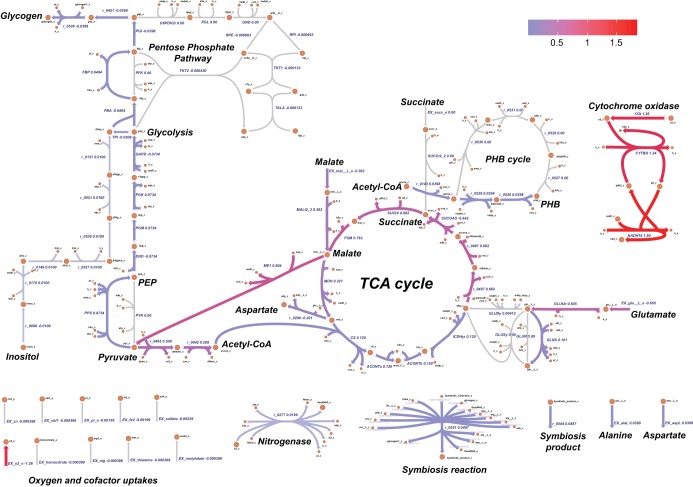
Flux distribution during symbiotic nitrogen fixation in *S. fredii*. Reactions are colored according to a scale of purple (flux of 0.04 mmol g^−1^ [dry weight] h^−1^) to red (flux of 1.8 mmol g^−1^ [dry weight] h^−1^ or greater). Reactions carrying zero flux or less than 0.04 mmol g^−1^ (dry weight) h^−1^ are colored gray. Colored arrowheads indicate the directionality of the flux. Flux metabolic networks were constructed using the Web tool Escher.

In symbiotic nitrogen fixation nodules, the host plant provides C_4_-dicarboxylic acids (malate or succinate) to bacteroids as the major energy source, while the microsymbiont returns organic fixation products to the host. The primary carbon source, malate, is transported into bacteroids to be metabolized through the tricarboxylic acid (TCA) cycle to provide reducing equivalents, energy, and precursors for biosynthetic pathways (12, 61, 62). The TCA cycle together with oxidative phosphorylation generates the energy and conditions required for the nitrogen fixation process. Oxygen levels must be maintained at the minimum to avoid inhibition of the nitrogenase activity, since it acts as a regulator of nitrogen fixation genes (*nif* and *fix* genes) (12, 20, 63). To examine the extent to which the model can capture these metabolic characteristics, flux balance analysis (FBA) was performed by maximizing the symbiosis reaction with malate as the primary carbon source and succinate and inositol as secondary carbon sources, with a small amount of oxygen available (maximum uptake rates being constrained by experimental data) (see Table S1). Figure 3 describes the flux distribution predicted by the model. In detail, the simulated exchange profile of the model shows the active uptake of glutamate and cofactors (including homocitrate, molybdenum, thiamine, cobalamin, zinc, iron, *myo*-inositol, phosphate, magnesium, and sulfate) as expected from the formulation of the symbiosis reaction (62, 64, 65). Oxygen is utilized by cytochrome oxidase to generate ATP. Ammonia, alanine, and aspartate are transported to the plant cell cytosol (39, 87). In addition, the predicted internal flux distribution describes the metabolic route of malate. Malate is actively transported into the cell and converted to pyruvate and CO_2_ via malate dehydrogenase. Then, pyruvate is decarboxylated by pyruvate dehydrogenase to form acetyl coenzyme A (acetyl-CoA) and enters the TCA cycle. The full oxidative TCA cycle is utilized by the bacteroid during the process, according to the model predictions (nonzero fluxes confirmed by flux variability analysis). Enzymes participating in the TCA cycle appear to have an active role in the nitrogen fixation process in *S. fredii*. TCA cycle mutants of S. meliloti, Rhizobium tropici, and Rhizobium leguminosarum have the common phenotype of being unable to fix nitrogen (68–70). The model, predicting the use of malate to produce energy and products for symbiosis through the TCA cycle, is therefore able to capture this important aspect of rhizobium-legume symbiosis. However, the role of the TCA cycle varies among rhizobia, with high metabolic plasticity observed in Bradyrhizobium japonicum (71, 72). PHB biosynthesis and gluconeogenesis pathways are active according to the simulation results and the definition of the symbiosis reaction. The use of these pathways is validated by the fact that both of these carbon storage compounds have been found to be present in determinate nodules of soybean (31–34). The model also predicts a low but nonzero flux through some reactions of the pentose phosphate pathway. This pathway generates ribose 5-phosphate, which is a precursor of purines and pyrimidines. Reduced symbiotic properties have been observed in pyrimidine and purine mutants of *S. fredii* (42, 43, 46–49). According to our reconstruction formulation and *in silico* results, the ammonia exported to the plant cell cytosol originates from the ammonia produced by the nitrogenase reaction (Fig. 3).

### Identification of genes involved in symbiosis.

Next, the model was used to identify symbiotic genes under standard nitrogen fixation conditions. As defined by the gene-protein-reaction (GPR) association, a gene was classified as a symbiotic gene if one or more reactions associated with that gene need to carry a nonzero flux to satisfy the symbiotic objective function. Results of simulations were categorized according to the effect of the gene deletion compared to that of the wild-type strain (see Table S2). According to the simulations, 141 of 541 genes (26%) were predicted to affect the symbiotic process, wherein 121 genes were classified as essential and 20 genes as having a partial effect, where their deletions resulted in a reduction in the capacity of *S. fredii* to fix atmospheric nitrogen. Most of these genes (84%) were located in the chromosome, while the others were present in the symbiosis plasmid, accessory plasmids, and chromid (Table S2). Coexpression patterns in the replicon of *S. fredii* strains have been observed under both free-living and symbiotic conditions (17).

10.1128/mSystems.00516-19.3 TABLE S2 Predicted symbiotic rate ratios between single-gene deletion mutants and wild-type strain. Download Table S2, XLSX file, 0.1 MB. Copyright © 2020 Contador et al. 2020 Contador et al. This content is distributed under the terms of the Creative Commons Attribution 4.0 International license.

Many genetic studies have been conducted to examine the legume-rhizobium symbiosis, providing important insights into the symbiotic capacity of rhizobia. A detailed comparative genomic study of rhizobia that nodulate soybean was used to validate the predicted symbiotic genes from our model (16). Genes predicted to affect the symbiotic process were compared to a list of known symbiosis genes. The analysis matched 50 of the 141 predicted symbiotic genes to previously identified genes in the literature. Based on our model, mutations in these genes had a negative impact on the symbiotic phenotypes of rhizobial strains. For example, symbiotic genes related to oxidative phosphorylation were identified. According to the simulation in our model, oxidative phosphorylation must be active *in vivo* to effectively fix nitrogen, as previously identified by pathway analysis (63). A complete list of these genes and the references providing the corresponding validating evidence are available in Table S3. Among the 91 remaining predicted symbiotic genes, 58 are orthologs to essential genes identified by *i*HZ565 (24). These genes have already been listed in the Database of Essential Genes (DEG) (73) and therefore have been shown to perform core functions that are required under both free-living and symbiotic conditions. The remaining 33 genes have not been shown to be essential to the symbiotic process. Their locations and predicted functions are provided in Table S2. Most of these genes are linked to the purine, pyrimidine, TCA, and amino acid pathways. One example is the gene AB395_00003474 (*sucB*). *sucB* and *sucA* encode subunits of the α-ketoglutarate dehydrogenase which catalyzes the conversion of α-ketoglutarate to succinyl-CoA in the TCA cycle. R. leguminosarum mutants form ineffective nodules (69). However, B. japonicum *sucA* mutants only showed a delay in the development of bacteroids, but their nitrogen fixation rates were similar to those of the wild type (72). On the other hand, AB395_00003483, which encodes a succinate dehydrogenase flavoprotein subunit, was predicted here to be a symbiotic gene. A gene encoding succinate dehydrogenase A (*sdhA*) has been identified as essential in *Bradyrhizobium* sp. strain ORS278 (74). Another symbiotic gene predicted by our model, AB395_00004794, is involved in the isoleucine and valine biosynthesis pathway. Tn*5* experiments have successfully identified this pathway as being related to the symbiotic process in *S. fredii* HH303, but the genes have not been characterized (46). The predicted gene is an *ilv* gene that encodes a dihydroxy-acid dehydratase. A BLASTP search of the amino acid sequence of the dihydroxy-acid dehydratases from S. meliloti 1021 (NP_384214, NP_436655, and NP_386934) against the *S. fredii* genome revealed identities of 89.37%, 64%, and 31.67%, respectively, with AB395_00004794 at the amino acid level. Mutations of *ilv* genes have been reported to result in phenotypes ranging from no nodule formation (Nod^−^) (35) to being unable to fix nitrogen (Nod delayed, Fix^−^) (75). In addition, genes related to the transport of cofactors such as sulfate were also predicted as novel symbiotic genes by our model. Additional experiments are required to confirm the functions and relevance of these genes in the TCA cycle, branched-amino-acid biosynthesis, and transport systems to the symbiotic processes in *S. fredii*. As stated above, *myo*-inositol uptake was active according to the model predictions. Simulations showed a slight reduction in the fixation rate, and shadow price analyses indicated it was a rate-limiting substrate. This is qualitatively but not quantitatively consistent with a previous finding that a mutation in the *myo*-inositol dehydrogenase gene (*idhA*) caused a much more significant reduction in the nitrogen fixation rate (66). Then, false-negative cases were evaluated. These cases represent discrepancies with experimentally observed phenotypes and indicate potential errors or knowledge gaps in the model. The symbiotic genes predictions exhibited 45 (8%) false negatives. The list of these genes can be found in Table S4. Analysis of these genes revealed active roles in processes such as nodulation, bacteroid development, microoxic respiration regulation, osmoregulation, and the inositol pathway. Regulation processes and steps previous to the symbiotic stage (e.g., root nodule formation) were not included in the current scope of the model, which explains this result. Additional false-negative cases occurred with knockout of genes involved in inositol catabolism, including *iolA* (AB395_0000388), *iolC* (AB395_00002655), and *iolD* (AB395_00002654) (76, 77). Shadow price analyses identified inositol as a rate-limiting step as stated above. *pstC* (AB395_0000141) and *pit* (AB395_00003896) involved in phosphate transport were classified as nonessential (78). FBA may use any transport reaction in the network to fix nitrogen under the standard nitrogen fixation condition. Additional constraints are required to simulate phosphate transport via high- and low-affinity systems.

10.1128/mSystems.00516-19.4 TABLE S3 List of predicted genes that influence symbiosis and the corresponding references for the associated experimental evidence. Download Table S3, DOCX file, 0.1 MB. Copyright © 2020 Contador et al. 2020 Contador et al. This content is distributed under the terms of the Creative Commons Attribution 4.0 International license.

10.1128/mSystems.00516-19.5 TABLE S4 List of false-negative predicted genes by the model. Download Table S4, DOCX file, 0.1 MB. Copyright © 2020 Contador et al. 2020 Contador et al. This content is distributed under the terms of the Creative Commons Attribution 4.0 International license.

### Nitrogen fixation capacity of *S. fredii* bacteroids in wild versus in cultivated soybean.

**FIG 4:**
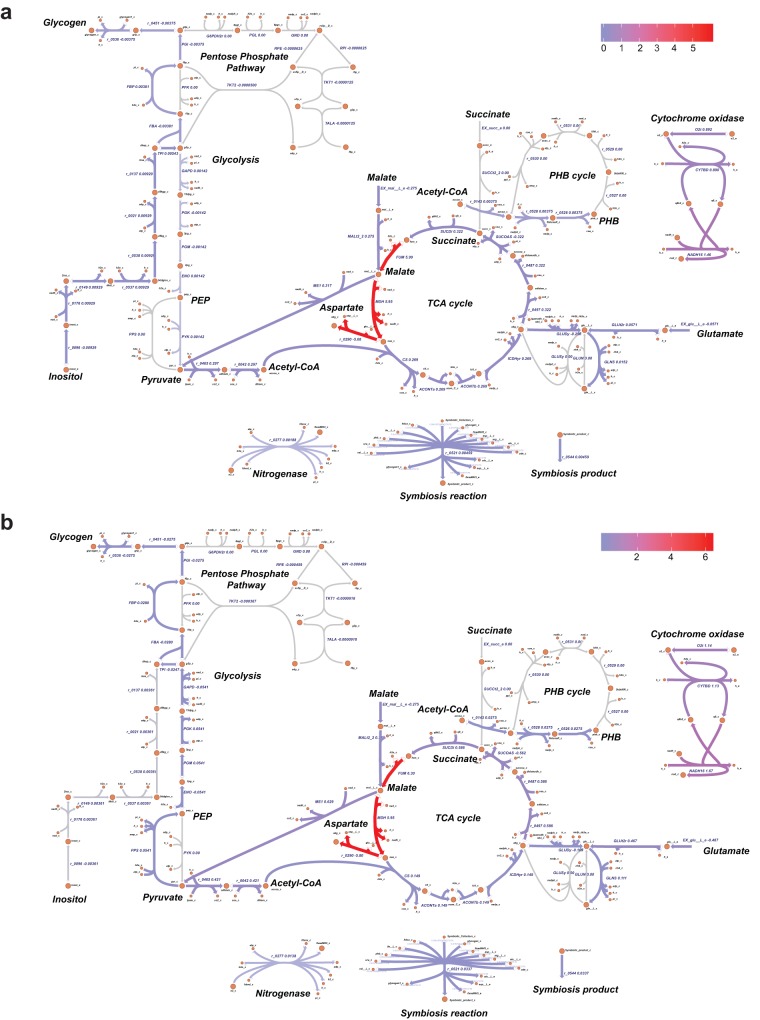

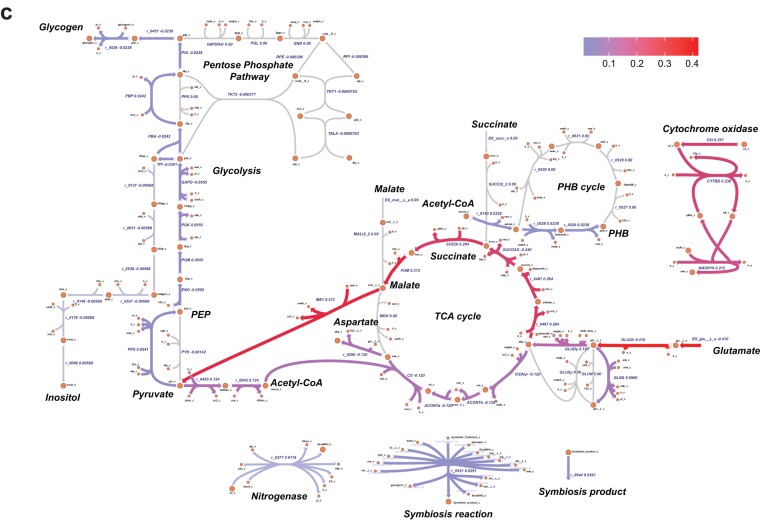
Nitrogen fixation capacity of *S. fredii* CCBAU45436 in wild soybean accession W05 (a) and cultivated soybean accession C08 (b). (c) The difference between the flux distributions of C08 and W05. Flux metabolic networks were constructed using the Web tool Escher. Reactions are colored according to a scale of purple (median flux or greater) to red (maximum flux). Colored arrowheads indicate the directionality of the flux. Reactions carrying zero flux or less than the median are colored gray.

**FIG 5:**
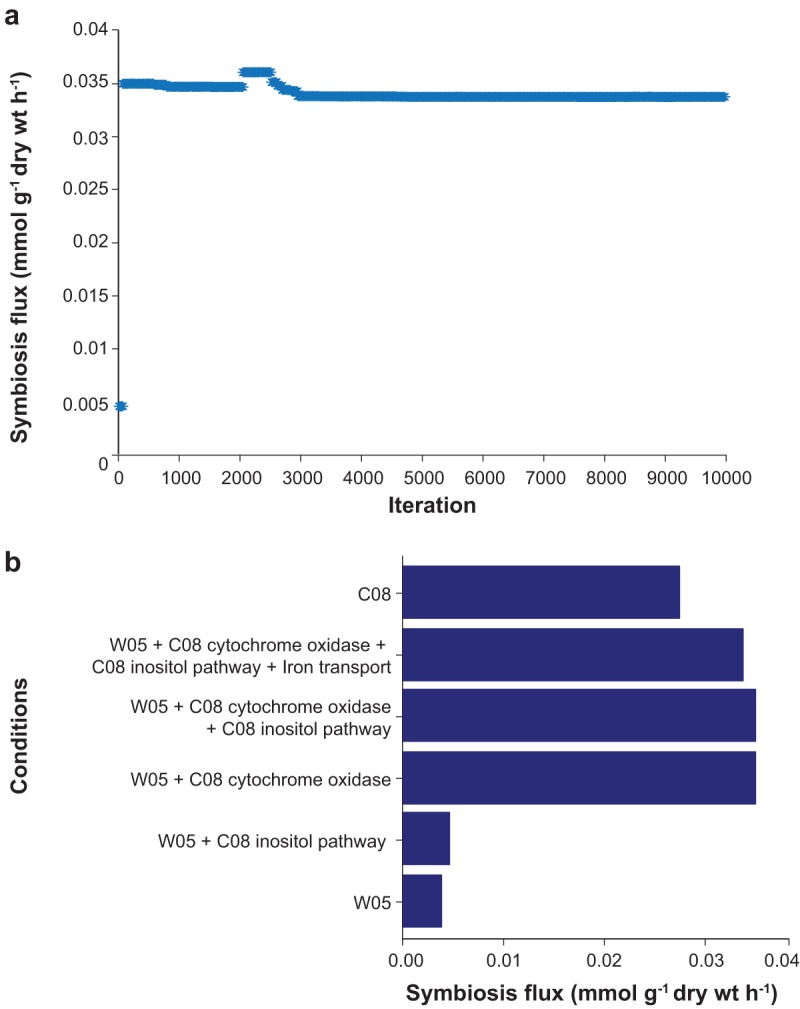
Evolutionary sensitivity analysis. (a) Symbiosis fluxes were determined by simulations starting from the W05 condition where bounds of the C08 condition were randomly imposed. (b) Reactions were identified where *S. fredii* inoculated in cultivated soybean outperformed that in wild soybean in nitrogen fixation.

Finally, transcriptome profiles were used to determine the nitrogen fixation capacity of CCBAU45436 with two different soybean cultivars, the wild soybean accession W05 and the cultivated soybean accession C08. Condition-specific models were built from *i*CC541 using the E-Flux approach (79). Gene expressions were used to constrain reaction flux bounds to assess the impact of the host plant on nitrogen fixation. Tables S5 and S6 list the flux ranges for each condition and raw expression data, respectively. Flux distributions are shown in Fig. 4. Exchange profiles of the models showed carbon and essential nutrient requirements. Additionally, certain secretion patterns overlap one another. As expected, malate is transported inside the cell and pyruvate is generated by the reaction catalyzed by malic enzyme. Pyruvate is further decarboxylated by pyruvate dehydrogenase to form acetyl-CoA. In both soybean cultivars, soybean bacteroid diverts acetyl-CoA into the TCA cycle and into the production of the lipid-like polymer PHB, both of which are pathways known for electron allocation in nitrogen-fixing bacteroids (80). Although similitudes are observed in the flux distributions, there are also differences in the use of some reactions and/or their magnitudes (Fig. 4c). To fully assess the impact on nitrogen fixation capacity, yields were calculated based on the model predictions (Table 5); 0.0032 and 0.0097 g symbiosis product per mmol of carbon uptake were obtained in the symbiosis with W05 and C08, respectively. Additionally, there was a difference in the flux of fixed ammonia with 0.0038 and 0.0275 mmol g^−1 ^(dry weight) h^−1^ transported from the bacteroid to W05 and C08, respectively. Simulations predicted a higher nitrogen fixation capacity of CCBAU45436 with C08 than with W05. C/N ratios of 0.1435 and 0.0426 were calculated for the C08 and W05 symbiotic conditions, respectively. The ammonia produced by nitrogen fixation in bacteroids is transported back to the host plant and assimilated into glutamate and glutamine by glutamine synthetase and glutamate synthase (20). In determinate nodules, such as those in soybean roots, glutamine enters the purine biosynthetic pathway to be further converted to ureides, allantoin, and allantoic acid (65, 81). Ureides, products of nitrogen fixation, are then transported from the root to the shoot. Thus, a higher ureides and nitrogen content should be expected in the root nodules of C08 compared to those of W05 when soybean is inoculated with CCBAU45436. Ureides and nitrogen content in root nodules are commonly used as an indicator of nitrogen fixation capacity and were previously investigated by our group using the same symbiotic conditions, with W05 and C08 being inoculated with *S. fredii* CCBAU45436. The results corroborate this model prediction (21). Experimental observations showed cultivated soybean outperformed wild soybeans on all the tested nitrogen fixation-related traits, in particular, the total nitrogen and total ureides accumulation (21). An imbalance in carbon and nitrogen metabolism in wild soybean was suggested as the cause of the lower capacity for nitrogen fixation. This experimental evidence validates the model predictions and indicates a consequence of the process of domestication and human selection in soybean. Furthermore, a simple evolutionary sensitivity analysis was performed to identify possible reactions (Fig. 4c) responsible for the higher performance of *S. fredii* with the cultivated soybean, by starting from the W05 condition (i.e., the model with flux bounds inferred from the E-Flux method used in the previous analysis), iteratively imposing bounds for the C08 condition randomly and fixing the bounds that increase nitrogen fixation until no further increase could be achieved (Fig. 5a). Across 10,000 simulations, different activity levels in the inositol pathway, cytochrome oxidase reaction, and iron transport were consistently identified to be the determinants for the predicted phenotypes (Fig. 5b). The inositol pathway is a lower-yielding pathway having a marginal effect on improving nitrogen fixation under the W05 condition (increased by 20%), while the cytochrome oxidase reaction appears to be a major bottleneck. However, a gene encoding an ATP-binding cassette (ABC)-type metal ion transport system (AB395_00003450) seemed to limit the nitrogen fixation rate under the C08 condition because of its lower expression level under the C08 condition than the W05 condition. Potential explanations for this include the existence of regulatory or resource allocation constraints that are not accounted for by the model. The proportionality assumption between maximum enzymatic activities and transcript levels applied to the model can serve as a proxy for estimating relative activities across conditions in the absence of additional data, but it is probably an oversimplification and may underestimate the activity of the metal ion transport reaction under the C08 condition or vice versa. Further *in vitro* and *in vivo* testing of these potential targets will help resolve this. Overall, a fine tuning of the energy, reducing factors, and regulations of gene expression are required to optimize the nitrogen fixation process.

**TABLE 5:** Comparison of nitrogen fixation capacity of *S. fredii* with W05 and C08 soybeans

Condition	Symbiosis flux yield (g mmol^−1^ carbon)	Fixed NH_3_ (mmol g^−1^ [dry wt] h^− 1^)	C/N
W05	0.0032	0.0038	0.0426
C08	0.0097	0.0275	0.1435

10.1128/mSystems.00516-19.6 TABLE S5 Upper and lower boundaries of each reaction in *i*CC541 of bacteroids isolated from nodules of *G. soja* (W05) and *G. max* (C08) as determined by gene expression data. Download Table S5, XLSX file, 0.1 MB. Copyright © 2020 Contador et al. 2020 Contador et al. This content is distributed under the terms of the Creative Commons Attribution 4.0 International license.

10.1128/mSystems.00516-19.7 TABLE S6 Average RPKM values of each gene in *i*CC541 under both symbiotic conditions (bacteroids isolated from *G. soja* W05 and *G. max* C08). Download Table S6, XLSX file, 0.1 MB. Copyright © 2020 Contador et al. 2020 Contador et al. This content is distributed under the terms of the Creative Commons Attribution 4.0 International license.

## DISCUSSION

In this study, we developed a metabolic model for the fast-growing Sinorhizobium fredii strain CCBAU45436. This represents the first metabolic reconstruction of the symbiotic form of *S. fredii* for exploring the metabolic capabilities of this strain in symbiosis with soybean, one of the most important food crops worldwide. This legume-rhizobium association can fix atmospheric nitrogen, which can be a great benefit to sustainable agriculture. A symbiosis equation was formulated to capture and describe the unique physiological characteristics of this interaction and the symbiotic nitrogen fixation. Each host plant-rhizobium interaction as well as their metabolic interactions are association specific. Extensive literature reviews must be conducted to include the distinctive characteristics that define a specific interaction, such as amino acids, nitrogenous bases, and cofactor requirements, since general rules have proved to be not universally applicable. All the current metabolic models available for rhizobia define a distinct symbiosis reaction. The establishment of this reaction is relevant to the accurate description of the biological processes, and new reconstructions should revise, adapt, or propose metabolic requirements as this area of research progresses. The symbiosis reaction must describe symbiotic phenotypes supporting nitrogen fixation, and the reaction should be modified if new experimental evidence shows that the phenotypes are a consequence of the rhizobia being unable to differentiate or infect the host plant. The symbiosis reaction of *S. fredii* described in this model, *i*CC541, allowed us to mimic symbiotic nitrogen fixation and bacteroid physiology in a way that is consistent with experimental data. Specifically, our model predicts the utilization of important pathways involved in nitrogen fixation, such as the TCA cycle, oxidative phosphorylation, gluconeogenesis, and PHB. Additionally, the effects of single-gene deletions on nitrogen fixation helped us identify the essential genes, with 77% of them in agreement with experimental evidence. Thirty-three novel symbiotic genes were predicted and can be useful as targets for selecting better performing rhizobium strains. False negatives gave insight about the missing regulatory factors and scope of the reconstruction. Functional differences can be identified using metabolic networks by examining cellular metabolism under different conditions. Taking advantage of the flexibility to integrate high-throughput data in the constraint-based modeling approach, transcriptomic data were used to assess nitrogen fixation capacity in wild and cultivated soybean populations inoculated with *S. fredii* CCBAU45436. Condition-specific models were used to predict and analyze symbiotic capabilities. The contextualized models predicted a higher performance of cultivated soybean compared to that of wild soybean under symbiosis. In addition, the inositol pathway and cytochrome oxidase reaction were identified as potential targets for strain design experiments. Evidence indicates that artificial selection during the domestication of soybean had a key role in the performance of bacteroids (21). In the future, new findings can be used to modify the reconstruction and adapt it to better represent the microsymbiont, such as experimental assessment of the ATP requirements for maintaining the bacteroid to further tune the model. Proteomic data of the *S. fredii* bacteroid would also help to complement and further improve the predictions made here with the model.

*i*CC541 only covers the metabolism of the bacteroid, the differentiated form of *S. fredii*, which is able to fix biological nitrogen for the host plant. As the next step, *i*CC541’s metabolic pathways should be extended to represent the free-living form of the rhizobium to study other niche conditions. Bulk soil and rhizosphere are part of the life cycle of rhizobia, and they must be colonized to induce nodule formation and allow the final differentiation of rhizobia. All current metabolic reconstructions for rhizobia describe the symbiotic nitrogen fixation process with the subsequent metabolic exchange between the microsymbiont and its host plant (19, 22–25). However, only two of the reconstructions available on rhizobia cover the full spectrum of metabolism. For example, phenotypic microarrays have been successfully used to test the metabolic capabilities of rhizobia at the free-living stage (19, 25). Expanding the scope of the model will require additional experimental data for validation.

The number of metabolic reconstructions is increasing as well as the number of their applications (82). At present, no minimum standards for publishing metabolic reconstructions have been clearly defined. This makes it difficult to assess quality and undermines reproducibility and transferability. New tools have been developed in the modeling community to solve this problem, and their use should be encouraged during the peer review process (26). This requirement will help to improve the usage of available models by both experts and nonexperts alike, which is one of the main goals for the development of new reconstructions. Also, the use of these tools will help with the curation of larger reconstructions. A snapshot report of our *S. fredii* reconstruction has been included in this study to help future users to understand, adapt, or improve the current reconstruction.

In summary, *i*CC541 provides a framework for studying the symbiotic nitrogen fixation capabilities of *S. fredii* within different host plants.

## MATERIALS AND METHODS

### Metabolic network reconstruction.

To create a metabolic reconstruction to represent the soybean microsymbiont *S. fredii*, the genome sequence of CCBAU45436 was used to identify orthologs. The genomes of six closely related rhizobial strains with high-quality draft genomes were selected to build an ortholog table. Ortholog predictions of rhizobia against *S. fredii* CCBAU45436 were identified by comparing two proteomes using KBase narrative interface methods (83). A minimum suboptimal best bidirectional hit (BBH) ratio of 90% was used. The ortholog table is available in Table S7 in the supplemental material.

10.1128/mSystems.00516-19.8 TABLE S7 List of orthologs of predicted symbiosis-related soybean genes used to build *i*CC541. The values represent best bidirectional hits (BBHs) percent. Download Table S7, XLSX file, 0.4 MB. Copyright © 2020 Contador et al. 2020 Contador et al. This content is distributed under the terms of the Creative Commons Attribution 4.0 International license.

Reactions from previously generated metabolic reconstructions of Sinorhizobium meliloti (19, 24) were added to *i*CC541 (model in this study) according to the ortholog table. Only reactions from *i*GD1575 (published model constructed on S. meliloti) associated with the symbiotic process were considered (19). Biomass and associated reactions of *i*GD1575 were not considered. Reactions associated with missing orthologs were not included in the preliminary draft. Gene-protein-reaction (GPR) associations were rewritten for each reaction based on the identified orthologs. GPR associations provide a link between different levels of information. Specifically, they indicate which protein is encoded by each gene and link the functional proteins to one or more enzymatic reactions. Additional reactions and gene contents from the Kyoto Encyclopedia of Genes and Genomes (KEGG) database (28) were added using the ortholog table. KEGG has no information related to *S. fredii* CCBAU45436, but it contains other rhizobium strains such as *S. fredii* NGR234 and HH103. In addition, reactions from a draft model automatically generated using KBase (29) were evaluated and added to the reconstruction. The poly-3-hydroxybutyrate (PHB) synthesis pathway was extracted from the *i*OR363 model constructed on Rhizobium etli (22). The draft reconstruction was manually inspected, curated, and validated to include main metabolic pathways related to the stages of symbiotic nitrogen fixation and to permit the computation of steady-state properties. An established reference protocol for the reconstruction of metabolic networks was followed throughout the process to maintain the consistency of the model formulation (67).

Literature and databases (e.g., KEGG, UniProtKB, Brenda, and NCBI) were used to obtain information for gene annotations, carbon-nitrogen pathway utilization, and other physiological and phenotypic properties during the stages of nitrogen fixation. GenBank locus tags were used as gene identifiers (IDs). Biochemical, Genetic, and Genomic (BiGG) and MetaNetX databases were used to standardize metabolite identifiers (58, 84). Lists of the reactions and metabolites included in the reconstruction are available in Tables S1 and S8. All genome sequences were downloaded from GenBank (85) on 20 July 2018. A published protocol was used to guarantee mass and charge balances (57). Briefly, the element balance of internal reactions was checked, including that of the symbiosis reaction. Inconsistencies were solved by finding the set of minimal formulae to solve the metabolites with unknown molecular groups and the metabolites with generic side groups. Elemental hydrogen balances were achieved through the addition of missing protons. ATP cycles were solved by checking the reversibility constraints.

10.1128/mSystems.00516-19.9 TABLE S8 List of metabolites included in the reconstruction of iCC541, with their corresponding charges, chemical formulae, and reference IDs in various databases Download Table S8, XLSX file, 0.1 MB. Copyright © 2020 Contador et al. 2020 Contador et al. This content is distributed under the terms of the Creative Commons Attribution 4.0 International license.

### Symbiosis reaction.

A symbiosis reaction was designed to represent the specific legume-rhizobium association studied in this work. Essential components were determined from the literature, and their fractional contributions were adapted from a previously published study (24). A biomass equation was not added to the reconstruction, since growth-associated pathways are switched off in nitrogen-fixing bacteroids (33). The symbiosis reaction and publications supporting its formulation are provided in Tables S1 and S9, respectively. The chemical formulae of symbiotic components were scaled to ensure that the symbiotic product produced by the model has a molecular weight (MW) of 1 g mmol^−1^ (57).

10.1128/mSystems.00516-19.10 TABLE S9 List of references used to formulate the symbiosis reactions. Download Table S9, DOCX file, 0.1 MB. Copyright © 2020 Contador et al. 2020 Contador et al. This content is distributed under the terms of the Creative Commons Attribution 4.0 International license.

### *In silico* symbiotic conditions.

Flux balance analysis (FBA) simulations (27) were performed to predict nitrogen fixation rates and metabolite productions by *S. fredii* in soybean nodule cells. The symbiosis equation was used as the objective function of the optimization problem assuming steady-state conditions. Predictions were matched qualitatively to the experimental results reported in the literature.

Legume-rhizobium symbiosis involves the exchange of metabolites and cofactors to obtain biological nitrogen. Metabolism and transport in bacteroids have been reviewed in detail (20, 51). The exchange of nutrients was simulated by allowing an unlimited uptake of phosphate, sulfate, cobalamin, molybdate, thiamine, iron, N_2_, H^+^, H_2_O, Mg^2+^, and Zn^2+^. The lower bounds of the respective exchange reactions were set to −1,000 mmol g^−1^ (dry weight) h^−1^. Uptake rates of C_4_-dicarboxylate acids supplied by the host plant, i.e., malate and succinate, were set to 1.44 mmol g^−1^ (dry weight) h^−1^ and 1.38 mmol g^−1 ^(dry weight) h^−1^, respectively (86). Oxygen consumption rate was limited to 1.26 mmol g^−1^ (dry weight) h^−1^ (87) to mimic the low-oxygen environment provided by root nodule cells (50). Lower bounds of glutamate and inositol were set according to a reference study (19). A flux of nongrowth-associated ATP maintenance (ATPm) was fixed at 4 mmol g^−1^ (dry weight) h^−1^ (25). Ammonia generated by the reaction catalyzed by the nitrogenase enzyme is exported to the plant cytoplasmic compartment (19). Nutrient and essential cofactor exchanges between the host plant and the bacteroid are schematically represented in Fig. 1.

The dual problem of the linear optimization described above was solved to determine the dual variables or shadow prices (88). Shadow prices measure the sensitivity of the objective function to changes in the availability of each metabolite. The symbiosis reaction was used as the objective function.

For the identification of symbiotic genes, *in silico* single-gene knockout simulations were performed by setting bounds of the corresponding reaction(s) to 0 mmol g^−1^ (dry weight) h^−1^ if a single gene was associated with one or multiple reactions (89). Reactions catalyzed by multiple noninteracting genes were not removed by a single-gene deletion. A symbiotic rate ratio between the deletion strain and wild type was computed to identify the set of essential genes for nitrogen fixation. A gene was classified as a symbiotic gene if the symbiotic rate ratio was less than the cutoff set at 0.05 (90). All simulations were performed using the MATLAB-based COBRA Toolbox (82). Gurobi Optimizer (v8.1.0) was employed as a linear programming solver. Escher was used for the visualization of the reconstruction and analysis (91). The E. coli core map was used as a template to build a map for *S. fredii* central metabolism. A model in JSON format was generated using COBRApy (92).

### Condition-specific models.

To evaluate the capacity of CCBAU45436 to fix atmospheric nitrogen in the nodules of different soybean cultivars, a transcriptome data set was incorporated into the analysis. Raw transcriptome sequencing (RNA-seq) data of *S. fredii* CCBAU45436 bacteroid at the symbiotic stage within the nodules of cultivated soybean C08 and wild soybean W05 were retrieved from a previous publication for reanalysis (17). In brief, the clean reads in fastq files were mapped to the reference genomes of *S. fredii* CCBAU45436 using HISAT2 (default parameters) (93). The counting of mapped reads for protein-coding genes from the sam files were performed by HTseq-count (94). The read counts were normalized with DESeq2 using the medians-of-ratios method (95). Gene expression data were preprocessed, and gene scores were calculated according to transcriptomic data integration methods (96, 97). The expression data were then used to tailor the metabolic reconstruction using the E-Flux approach (79). Gene scores were mapped to the model by parsing the GPR rules associated with each reaction (98). No gene expression data and orphan reactions were given an expression value of −1. Lower and upper bounds of the reactions were determined by flux variability analysis (99). The minimum percentage of the optimal solution was set to zero. Under each condition, i.e., W05 and C08, the minimum values between expression reactions were identified and bounds were scaled down by the corresponding expression ratio between W05 and C08 (100).

Flux distributions of bacteroids within the nodules of C08 and W05 were compared by simultaneously solving two FBA problems and minimize the 1-norm of the flux vectors in the MATLAB-based COBRA Toolbox (82). An evolutionary sensitivity analysis was performed to evaluate these flux distributions and identify which gene expression changes are critical under these two conditions. The condition with the lower yield (W05) was selected as the starting point (i.e., model imposed with the flux bounds inferred using the aforementioned E-Flux method). A reaction was randomly selected, and the flux bound was changed randomly toward the bound for the same reaction under the condition with higher yield (C08) until the resultant yield stabilized and reached the level under condition C08.

### Data availability.

The reconstruction, memote reports, and scripts are available at https://github.com/cacontad/SfrediiScripts.
