# Supplementary material for: Metabolic Analyses of Nitrogen Fixation in the Soybean Microsymbiont Sinorhizobium fredii Using Constraint-Based Modeling
Source: mSystems. 2020 Feb 18;5(1):e00516-19. doi: 10.1128/mSystems.00516-19 (PMC7029217; doi:10.1128/mSystems.00516-19)

Fig. S1 Snapshot reports obtained from memote for comparison of *i*CC541 with *S. meliloti* models in HTML format.

Consistency

Compared Models	iCC541.xml	iHZ565.xml	iGD1575.xml
Stoichiometric Consistency	100.0%	28.4%	67.5%
Mass Balance	99.6%	0.0%	0.0%
Charge Balance	98.6%	0.0%	97.1%
Metabolite Connectivity	100.0%	94.1%	96.9%
Unbounded Flux In Default Medium	0.0%	0.0%	0.0%
Sub Total	85%	26%	57%

Annotation - Metabolites

Compared Models	iCC541.xml	iHZ565.xml	iGD1575.xml
Presence of Metabolite Annotation	100.0%	0.0%	0.0%
Metabolite Annotations Per Database			Info
pubchem.compound	0.0%	0.0%	0.0%
kegg.compound	98.6%	0.0%	0.0%
seed.compound	100.0%	0.0%	0.0%
inchikey	0.0%	0.0%	0.0%
inchi	0.0%	0.0%	0.0%
chebi	0.0%	0.0%	0.0%
hmdb	0.0%	0.0%	0.0%
reactome	0.0%	0.0%	0.0%
metanetx.chemical	91.9%	0.0%	0.0%
bigg.metabolite	100.0%	0.0%	0.0%
biocyc	0.0%	0.0%	0.0%
Metabolite Annotation Conformity Per Database			Info
pubchem.compound	0.0%	0.0%	0.0%
kegg.compound	100.0%	0.0%	0.0%
seed.compound	99.2%	0.0%	0.0%
inchikey	0.0%	0.0%	0.0%
inchi	0.0%	0.0%	0.0%
chebi	0.0%	0.0%	0.0%
hmdb	0.0%	0.0%	0.0%

SBML

Compared Models	iCC541.xml	iHZ565.xml	iGD1575.xml
SBML Level and Version	SBML Level 3 Version 1	SBML Level 2 Version 1	SBML Level 2 Version 1
FBC enabled	true	false	false

Basic Information

Compared Models	iCC541.xml	iHZ565.xml	iGD1575.xml
Model Identifier	M_iCC541	Model	kb_g_570_fbamdl11
Total Metabolites	508	522	1,579
Total Reactions	538	526	1,825
Total Genes	541	566	1,577
Total Compartments	2	2	2
Metabolic Coverage	0.99	0.93	1.16

Metabolite Information

Compared Models	iCC541.xml	iHZ565.xml	iGD1575.xml
Unique Metabolites	508	522	1,311
Duplicate Metabolites in Identical Compartments	0	0	0
Metabolites without Charge	4	522	0
Metabolites without Formula	2	0	0
Medium Components	20	19	267

Reaction Information

Compared Models	iCC541.xml	iHZ565.xml	iGD1575.xml
Purely Metabolic Reactions	483	503	1,531
Purely Metabolic Reactions with Constraints	1	0	3
Transport Reactions	27	0	0
Transport Reactions with Constraints	0	0	0
Thermodynamic Reversibility of Purely Metabolic Reactions	0.35	1.00	1.00
Reactions With Partially Identical Annotations	0.90	0.00	0.00
Duplicate Reactions	0.00	0.00	0.00
Reactions With Identical Genes	0.50	0.49	0.70

reactome	0.0%	0.0%	0.0%
metanetx.chemical	100.0%	0.0%	0.0%
bigg.metabolite	98.4%	0.0%	0.0%
biocyc	0.0%	0.0%	0.0%
Uniform Metabolite Identifier Namespace	100.0%	99.0%	100.0%

Annotation - Reactions

Compared Models	iCC541.xml	iHZ565.xml	iGD1575.xml
Presence of Reaction Annotation	100.0%	0.0%	0.0%
Reaction Annotations Per Database			Info
rhea	0.0%	0.0%	0.0%
kegg.reaction	86.4%	0.0%	0.0%
seed.reaction	99.6%	0.0%	0.0%
metanetx.reaction	0.0%	0.0%	0.0%
bigg.reaction	100.0%	0.0%	0.0%
reactome	0.0%	0.0%	0.0%
ec-code	89.5%	0.0%	0.0%
brenda	0.0%	0.0%	0.0%
biocyc	0.0%	0.0%	0.0%
Reaction Annotation Conformity Per Database			Info
rhea	0.0%	0.0%	0.0%
kegg.reaction	100.0%	0.0%	0.0%
seed.reaction	90.5%	0.0%	0.0%
metanetx.reaction	0.0%	0.0%	0.0%
bigg.reaction	10.3%	0.0%	0.0%
reactome	0.0%	0.0%	0.0%
ec-code	100.0%	0.0%	0.0%
brenda	0.0%	0.0%	0.0%
biocyc	0.0%	0.0%	0.0%
Uniform Reaction Identifier Namespace	100.0%	99.0%	100.0%
Sub Total	69%	25%	25%

Gene-Protein-Reaction (GPR) Associations

Compared Models	iCC541.xml	iHZ565.xml	iGD1575.xml
Reactions without GPR	19	15	54
Fraction of Transport Reactions without GPR	0.22	1.00	1.00
Enzyme Complexes	71	56	

Biomass

Compared Models	iCC541.xml	iHZ565.xml	iGD1575.xml
Biomass Reactions Identified	0	0	2
Biomass Consistency			Info
biomass_bulk_c0			0.00
biomass_rhizo_c0			0.00
Biomass Production In Default Medium			Info
biomass_bulk_c0			328.79
biomass_rhizo_c0			328.76
Unrealistic Growth Rate In Default Medium			Info
biomass_bulk_c0			true
biomass_rhizo_c0			true
Biomass Production In Complete Medium			Info
biomass_bulk_c0			328.79
biomass_rhizo_c0			328.76
Blocked Biomass Precursors In Default Medium			Info
biomass_bulk_c0			4
biomass_rhizo_c0			4
Blocked Biomass Precursors In Complete Medium			Info
biomass_bulk_c0			4
biomass_rhizo_c0			4
Ratio of Direct Metabolites in Biomass Reaction			Info
biomass_bulk_c0			0.00
biomass_rhizo_c0			0.00
Number of Missing Essential Biomass Precursors			Info
biomass_bulk_c0			37
biomass_rhizo_c0			37

Annotation - Genes

Compared Models	iCC541.xml	iHZ565.xml	iGD1575.xml
Presence of Gene Annotation	100.0%	0.0%	0.0%
Gene Annotations Per Database			Info
refseq	0.0%	0.0%	0.0%
uniprot	0.0%	0.0%	0.0%
ecogene	0.0%	0.0%	0.0%
kegg.genes	0.0%	0.0%	0.0%
ncbigi	0.0%	0.0%	0.0%
ncbigene	0.0%	0.0%	0.0%
ncbiprotein	0.0%	0.0%	0.0%
ccds	0.0%	0.0%	0.0%
hprd	0.0%	0.0%	0.0%
asap	0.0%	0.0%	0.0%
Gene Annotation Conformity Per Database			Info
refseq	0.0%	0.0%	0.0%
uniprot	0.0%	0.0%	0.0%
ecogene	0.0%	0.0%	0.0%
kegg.genes	0.0%	0.0%	0.0%
ncbigi	0.0%	0.0%	0.0%
ncbigene	0.0%	0.0%	0.0%
ncbiprotein	0.0%	0.0%	0.0%
ccds	0.0%	0.0%	0.0%
hprd	0.0%	0.0%	0.0%
asap	0.0%	0.0%	0.0%
Sub Total	33%	0%	0%

Energy Metabolism

Compared Models	iCC541.xml	iHZ565.xml	iGD1575.xml
Non-Growth Associated Maintenance Reaction	1	1	1
Growth-associated Maintenance in Biomass Reaction			Info
biomass_bulk_c0			false
biomass_rhizo_c0			false
Number of Reversible Oxygen-Containing Reactions	4	4	18

Network Topology

Compared Models	iCC541.xml	iHZ565.xml	iGD1575.xml
Universally Blocked Reactions			
Orphan Metabolites	30	30	65
Dead-end Metabolites	37	35	87
Stoichiometrically Balanced Cycles			
Metabolite Production In Complete Medium	156	159	436
Metabolite Consumption In Complete Medium	276	238	572

Matrix Conditioning

Compared Models	iCC541.xml	iHZ565.xml	iGD1575.xml
Ratio Min/Max Non-Zero Coefficients	0.00	0.00	0.00
Independent Conservation Relations	39	66	53
Rank	466	456	1470
Degrees Of Freedom	69	70	355

Annotation - SBO Terms

Compared Models	iCC541.xml	iHZ565.xml	iGD1575.xml
Metabolite General SBO Presence	0.0%	0.0%	0.0%
Metabolite SBO:0000247 Presence	0.0%	0.0%	0.0%
Reaction General SBO Presence	100.0%	0.0%	0.0%
Metabolic Reaction SBO:0000176 Presence	99.8%	0.0%	0.0%
Transport Reaction SBO:0000185 Presence	77.8%	Skipped	Skipped
Exchange Reaction SBO:0000627 Presence	100.0%	0.0%	0.0%
Demand Reaction SBO:0000628 Presence	100.0%	Skipped	Skipped
Sink Reactions SBO:0000632 Presence	100.0%	Skipped	0.0%
Gene General SBO Presence	100.0%	0.0%	0.0%
Gene SBO:0000243 Presence	0.0%	0.0%	0.0%
Biomass Reactions SBO:0000629 Presence	Skipped	Skipped	0.0%
Sub Total	62%	0%	0%
Total Score	70%	14%	26%

Total Score

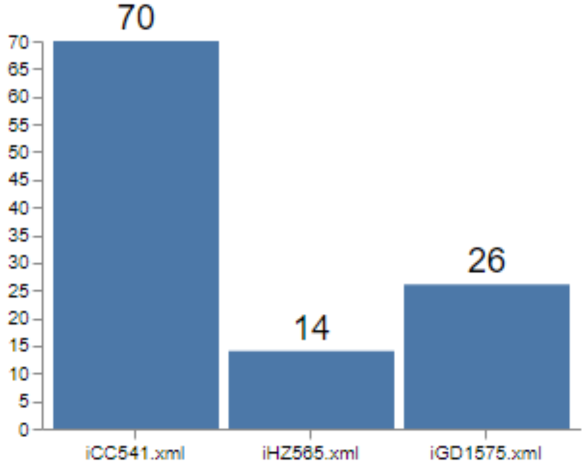

Score per Category

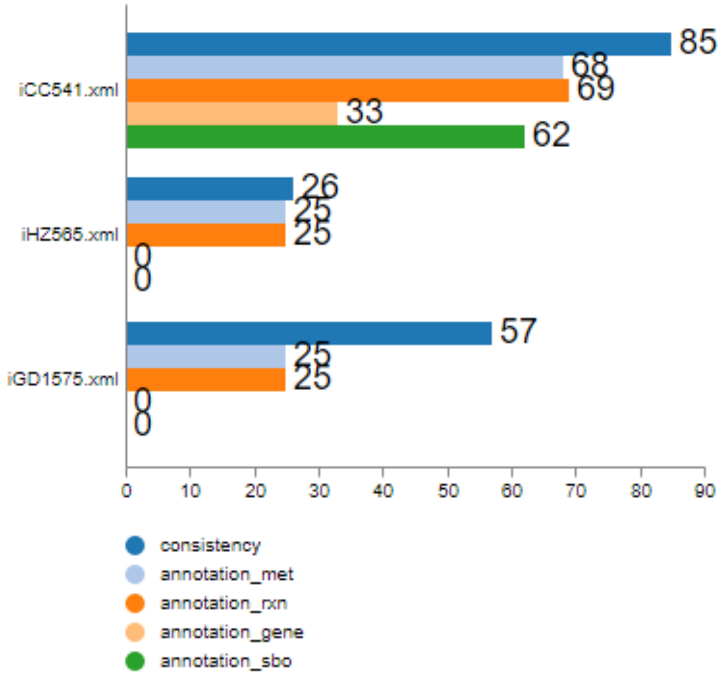

Supplement: FIG S1 [file mSystems.00516-19-sf001.pdf]
