# Supplementary material for: Metabolic Analyses of Nitrogen Fixation in the Soybean Microsymbiont Sinorhizobium fredii Using Constraint-Based Modeling
Source: mSystems. 2020 Feb 18;5(1):e00516-19. doi: 10.1128/mSystems.00516-19 (PMC7029217; doi:10.1128/mSystems.00516-19)
Supplement: TABLE S3 [file mSystems.00516-19-st003.docx]

**TableS3:** List of predicted genes that influence symbiosis.

| **GenBank ID** | **Gene** | **Product** | **Reference** |
| --- | --- | --- | --- |
| AB395_0000120 | *pyrC* | dihydroorotase | (1) |
| AB395_0000121 | *pyrE* | orotate phosphoribosyltransferase | (2) |
| AB395_00001214 | *pdhAa* | pyruvate dehydrogenase E1 component subunit alpha | (2) |
| AB395_00001215 | *pdhAb* | pyruvate dehydrogenase complex E1 component subunit beta | (2) |
| AB395_0000124 | *pgi* | glucose-6-phosphate isomerase | (3) |
| AB395_00001355 | *phbC* | class I poly(R)-hydroxyalkanoic acid synthase | (2) |
| AB395_00001559 | *purB* | adenylosuccinate lyase | (4) |
| AB395_00001587 | *nifS* | cysteine desulfurase | (2) |
| AB395_00001668 | *purQ* | phosphoribosylformylglycinamidine synthase subunit PurQ | (5) |
| AB395_00001672 | *purL* | phosphoribosylformylglycinamidine synthase subunit PurL | (5,6) |
| AB395_00001849 | *fixH* | cation transporter | (2) |
| AB395_00001850 | *fixG* | cytochrome c oxidase accessory protein CcoG | (2) |
| AB395_00001876 | *cobO* | cob(I)yrinic acid a,c-diamide adenosyltransferase | (7) |
| AB395_00002031 | *SMb20493* | short-chain dehydrogenase | (2) |
| AB395_00002043 | *ilvC* | ketol-acid reductoisomerase | (2) |
| AB395_00002092 | *ilvI* | acetolactate synthase 3 large subunit | (2) |
| AB395_0000210 | *rkpA* | beta-ketoacyl synthase | (2) |
| AB395_00003102 | *tkt2* | transketolase | (2) |
| AB395_00003154 | *hemH* | ferrochelatase | (2) |
| AB395_00003190 | *ilvD2* | dihydroxy-acid dehydratase | (2) |
| AB395_000032 | *dme* | NADP-dependent malic enzyme | (2) |
| AB395_00003206 | *pgm* | alpha-D-glucose phosphate-specific phosphoglucomutase | (2) |
| AB395_00003295 | *modA* | molybdate ABC transporter substrate-binding protein | (2) |
| AB395_00003296 | *modB* | molybdate ABC transporter permease subunit | (2) |
| AB395_00003450 | *sitA(mntA)* | metal ABC transporter substrate-binding protein | (2) |
| AB395_00003482 | *sdhB* | succinate dehydrogenase iron-sulfur subunit | (8) |
| AB395_00003619 | *phbB* | beta-ketoacyl-ACP reductase | (2) |
| AB395_00003620 | *phbA* | acetyl-CoA C-acetyltransferase | (2) |
| AB395_00004042 | *fabI2* | enoyl-[acyl-carrier-protein] reductase FabI | (2) |
| AB395_00004141 | *pyrF* | orotidine-5'-phosphate decarboxylase** | (9) |
| AB395_00004153 | *gshB* | glutathione synthase | (10) |
| AB395_00004446 | *nifB* | nitrogenase cofactor biosynthesis protein NifB | (2) |
| AB395_00004450 | *fixB* | electron transfer flavoprotein subunit alpha/FixB family protein | (2) |
| AB395_00004451 | *fixA* | protein FixA | (2) |
| AB395_00004462 | *nifD* | nitrogenase molybdenum-iron protein alpha chain | (2) |
| AB395_00004463 | *nifK* | nitrogenase molybdenum-iron protein subunit beta | (2) |
| AB395_00004465 | *nifN* | nitrogenase iron-molybdenum cofactor biosynthesis protein NifN | (2) |
| AB395_00004603 | *nifH* | nitrogenase iron protein | (2) |
| AB395_0000564 | *fabI1* | enoyl-[acyl-carrier-protein] reductase FabI | (2) |
| AB395_00005830 | *nodQ1* | adenylyl-sulfate kinase | (2) |
| AB395_00006012 | *fabG* | SDR family NAD(P)-dependent oxidoreductase | (2) |
| AB395_0000629 | *cysN* | sulfate adenylyltransferase subunit CysN | (11) |
| AB395_00001216 | *pdhB* | pyruvate dehydrogenase complex dihydrolipoamide acetyltransferase | (2) |
| AB395_0000838 | *nodG* | 3-oxoacyl-[acyl-carrier-protein] reductase | (2) |
| AB395_0000882 | *purM* | phosphoribosylformylglycinamidine cyclo-ligase | (12) |
| AB395_00003475 | *sucA* | 2-oxoglutarate dehydrogenase E1 component | (13) |
| AB395_00003478 | *mdh* | malate dehydrogenase | (2) |
| AB395_00003882 | *gpmA* | phosphoglycerate mutase | (14) |
| AB395_00004818 | *dctA* | dicarboxylate/amino acid:cation symporter** | (15) |
| AB395_00002682 | *sucD* | succinyl-CoA synthetase | (13) |

** gene annotation suggestions

**References**

1. Wang D, Wang YC, Wu LJ, Liu JX, Zhang P, Jiao J, Yan H, Liu T, Tian CF, Chen WX. Construction and pilot screening of a signature-tagged mutant library of *Sinorhizobium fredii*. Arch Microbiol. 2016;198(2):91–9.

2. Mao C, Qiu J, Wang C, Charles TC, Sobral BW. NodMutDB: a database for genes and mutants involved in symbiosis. Bioinformatics. 2005;21(12):2927–9.

3. Arias A, Cervenansky C, Gardiol A, Martinez-Drets. G. Phosphoglucose isomerase mutant of *Rhizobium meliloti*. J Bacteriol. 1979;137(1):409–14.

4. Okazaki S, Hattori Y, Saeki. K. The *Mesorhizobium loti* *purB* gene is involved in infection thread formation and nodule development in *Lotus japonicus*. J Bacteriol. 2007;189(22):8347–52.

5. Newman JD, Diebold RJ, Schultz BW, Noel KD. Infection of soybean and pea nodules by *Rhizobium* spp. purine auxotrophs in the presence of 5-aminoimidazole-4-carboxamide riboside. J Bacteriol. 1994;176(11):3286–94.

6. Buendía-Clavería AM, Moussaid A, Ollero FJ, Vinardell JM, Torres A, Moreno J, Gil-Serrano AM, Rodríguez-Carvajal MA, Tejero-Mateo P, Peart JL, Brewin NJ, Ruiz-Sainz JE. A *purL* mutant of *Sinorhizobium fredii* HH103 is symbiotically defective and altered in its lipopolysaccharide. Microbiology. 2003;149(7):1807–18.

7. Medina C, Crespo-Rivas JC, Moreno J, Espuny MR, Cubo MT. Mutation in the *cobO* gene generates auxotrophy for cobalamin and methionine and impairs the symbiotic properties of *Sinorhizobium fredii* HH103 with soybean and other legumes. Arch Microbiol. 2009;191(1):11–21.

8. Dymov SI, Meek DJJ, Steven B, Driscoll BT. Insertion of transposon Tn5tac1 in the *Sinorhizobium meliloti* malate dehydrogenase (*mdh*) gene results in conditional polar effects on downstream TCA cycle genes. Mol Plant Microbe Interact. 2004;17(12):1318–27.

9. Crespo-Rivas JC, Margaret I, Pérez-Montaño F, López-Baena FJ, Vinardell JM, Ollero FJ, Moreno FJ, Ruiz-Sainz JE, Buendía-Clavería AM. A *pyrF* auxotrophic mutant of *Sinorhizobium fredii* HH103 impaired in its symbiotic interactions with soybean and other legumes. Int Microbiol. 2007;10(3):169–76.

10. Harrison J, Jamet A, Muglia CI, Van de Sype G, Aguilar OM, Puppo A, Frendo P. Glutathione plays a fundamental role in growth and symbiotic capacity of *Sinorhizobium meliloti*. J Bacteriol. 2005;187(1):168–74.

11. Laeremans T, Martinez-Romero E, Vanderleyden J. Isolation and sequencing of a second *Rhizobium tropici* CFN299 genetic locus that contains genes homologous to amino acid sulphate activation genes. DNA Seq. 1998;9(1):65–70.

12. Stevens JB, Luca NG de, Beringer JE, Ringer JP, Yeoman KH, Johnston AWB. The purMN genes of *Rhizobium leguminosarum* and a superficial link with siderophore production. Mol Plant-Microbe Interact. 2000;13(2):228–31.

13. Walshaw DL, Wilkinson A, Mundy M, Smith M, Poole PS. Regulation of the TCA cycle and the general amino acid permease by overflow metabolism in *Rhizobium leguminosarum*. Microbiology. 1997;143:2209–21.

14. Bonaldi K, Gourion B, Fardoux J, Hannibal L, Cartieaux F, Boursot M, Vallenet D, Chaintreuil C, Prin Y, Nouwen N, Giraud E. Large-Scale Transposon Mutagenesis of Photosynthetic *Bradyrhizobium* Sp. Strain ORS278 Reveals New Genetic Loci Putatively Important for Nod-Independent Symbiosis with *Aeschynomene indica*. Mol Plant-Microbe Interact. 2010;23(6):760–70.

15. Udvardi MK, Price GD, Gresshoff PM, Day DA. A dicarboxylate transporter on the peribacteroid membrane of soybean nodules. FEBS Lett. 1988;231(1):36–40.
