# Supplementary material for: Metabolic Analyses of Nitrogen Fixation in the Soybean Microsymbiont Sinorhizobium fredii Using Constraint-Based Modeling
Source: mSystems. 2020 Feb 18;5(1):e00516-19. doi: 10.1128/mSystems.00516-19 (PMC7029217; doi:10.1128/mSystems.00516-19)
Supplement: TABLE S4 [file mSystems.00516-19-st004.docx]

**Table S4:** List of false negative predicted genes by the model.

| **GenBank ID** | **Gene** | **Product** | **Reference** |
| --- | --- | --- | --- |
| AB395_00001239 | *cysG* | uroporphyrinogen-III C-methyltransferase | (1) |
| AB395_0000125 | *fadD* | long-chain-fatty-acid-CoA ligase | (1) |
| AB395_00001281 | *gltA* | citrate (Si)-synthase | (1) |
| AB395_00001306 | *surE* | 5'/3'-nucleotidase SurE | (1) |
| AB395_0000141 | *pstC* | phosphate ABC transporter permease subunit PstC | (1) |
| AB395_00001443 | *trpC* | indole-3-glycerol phosphate synthase | (1) |
| AB395_00001444 | *trpD* | anthranilate phosphoribosyltransferase | (1) |
| AB395_00001448 | *nodM* | glutamine-fructose-6-phosphate transaminase (isomerizing) | (1) |
| AB395_0000145 | *phoB* | DNA-binding response regulator | (1) |
| AB395_0000153 | *metZ* | O-succinylhomoserine sulfhydrylase | (2) |
| AB395_00001854 | *fixP1* | cytochrome-c oxidase | (1,3) |
| AB395_00001855 | *fixQ1* | cbb3-type cytochrome c oxidase subunit 3 | (1,3) |
| AB395_00001856 | *fixO* | cytochrome-c oxidase | (1,3) |
| AB395_00001857 | *fixN1* | cytochrome-c oxidase | (1,3) |
| AB395_00002338 | *proC* | pyrroline-5-carboxylate reductase | (1) |
| AB395_00002405 | *leuA1* | 2-isopropylmalate synthase | (1) |
| AB395_00002411 | *trpE* | anthranilate synthase | (1) |
| AB395_00002429 | *opaB* | aspartate aminotransferase family protein | (4) |
| AB395_00002480 | *idhA* | inositol 2-dehydrogenase | (5) |
| AB395_00002645 | *fixP3* | cytochrome-c oxidase | (1,3) |
| AB395_00002646 | *fixQ3* | CcoQ/FixQ family Cbb3-type cytochrome c oxidase assembly chaperone | (1,3) |
| AB395_00002647 | *fixO3* | cytochrome-c oxidase | (1,3) |
| AB395_00002648 | *fixN3* | cytochrome-c oxidase | (1,3) |
| AB395_00002654 | *iolD* | 3D-(3,5/4)-trihydroxycyclohexane-1,2-dione acylhydrolase (decyclizing) | (6) |
| AB395_00002655 | *iolC* | 5-dehydro-2-deoxygluconokinase | (7) |
| AB395_00003205 | *glgA1* | glycogen synthase | (1) |
| AB395_00003394 | *metH* | methionine synthase | (8) |
| AB395_00003488 | *leuC* | 3-isopropylmalate dehydratase large subunit | (1) |
| AB395_00003508 | *leuD* | 3-isopropylmalate dehydratase small subunit | (1) |
| AB395_00003511 | *metA* | homoserine O-succinyltransferase | (1) |
| AB395_00003635 | *pyc* | pyruvate carboxylase | (1) |
| AB395_00003648 | *opaA* | aspartate aminotransferase family protein | (4) |
| AB395_00003669 | *leuB* | 3-isopropylmalate dehydrogenase | (1) |
| AB395_0000377 | *iolA* | methylmalonate-semialdehyde dehydrogenase (CoA acylating) | (7) |
| AB395_00003896 | *pit* | inorganic phosphate transporter | (1) |
| AB395_0000416 | *guaB* | IMP dehydrogenase | (9) |
| AB395_00004475 | *y4wE* | histidinol-phosphate transaminase | (10) |
| AB395_00004576 | *y4xP* | cysteine synthase family protein | (11) |
| AB395_00005828 | *asnO* | asparagine synthase (glutamine-hydrolyzing) | (1) |
| AB395_00005927 | *glgA2* | glycogen synthase | (1) |
| AB395_00006220 | *thiC* | phosphomethylpyrimidine synthase | (1) |
| AB395_00006694 | *arcB* | ornithine carbamoyltransferase | (12) |
| AB395_0000765 | *relA* | bifunctional (p)ppGpp synthetase/guanosine-3',5'-bis(diphosphate) 3'-pyrophosphohydrolase | (1) |
| AB395_0000818 | *psd* | phosphatidylserine decarboxylase family protein | (1) |
| AB395_0000912 | *glyA* | serine hydroxymethyltransferase | (1) |

**References**

1. Mao C, Qiu J, Wang C, Charles TC, Sobral BW. NodMutDB: a database for genes and mutants involved in symbiosis. Bioinformatics. 2005;21(12):2927–9.

2. Taté R, Riccio A, Caputo E, Iaccarino M, Patriarca EJ. The *Rhizobium etli metZ* Gene Is Essential for Methionine Biosynthesis and Nodulation of *Phaseolus vulgaris*. Mol Plant-Microbe Interact. 1999;12(1):24–34.

3. Preisig O, Anthamatten D, Hennecke H. Genes for a microaerobically induced oxidase complex in *Bradyrhizobium japonicum* are essential for a nitrogen-fixing endosymbiosis. Proc Natl Acad Sci. 1993;90(8):3309–13.

4. Prell J, Bourde A, Karunakaran R, Lopez-Gomez M, Poole P. Pathway of gamma-aminobutyrate Metabolism in *Rhizobium leguminosarum* 3841 and Its Role in Symbiosis. J Bacteriol. 2009;191(7):2177–86.

5. Jiang G, Krishnan AH, Kim Y, Wacek TJ, Krishnan HB, Acteriol JB. A Functional myo -Inositol Dehydrogenase Gene Is Required for Efficient Nitrogen Fixation and Competitiveness of *Sinorhizobium fredii* USDA191 To Nodulate Soybean (*Glycine max* [ L.] Merr.). J Bacteriol. 2001;183(8):2595–604.

6. Fry J, Wood M, Poole PS. Investigation of myo-Inositol Catabolism in *Rhizobium leguminosarum* bv. *viciae* and Its Effect on Nodulation Competitiveness. Mol Plant-Microbe Interact. 2001;14(8):1016–25.

7. Kohler PRA, Zheng JY, Schoffers E, Rossbach S. Inositol Catabolism, a Key Pathway in *Sinorhizobium meliloti* for Competitive Host Nodulation. Appl Environ Microbiol. 2010;76(24):7972–80.

8. Jiang JQ, Wei W, Du BH, Li XH, Wang L, Yang SS. Salt-tolerance genes involved in cation efflux and osmoregulation of *Sinorhizobium fredii* RT19 detected by isolation and characterization of Tn5 mutants. FEMS Microbiol Lett. 2004;239:139–46.

9. Collavino M, Riccillo PM, Grasso DH, Crespi M, Aguilar OM. GuaB Activity Is Required in *Rhizobium tropici* During the Early Stages of Nodulation of Determinate Nodules but Is Dispensable for the *Sinorhizobium meliloti* – Alfalfa Symbiotic Interaction. Mol Plant-Microbe Interact. 2005;18(7):742–50.

10. Theunis M, Kobayashi H, Broughton WJ, Prinsen E. Flavonoids, NodD1, NodD2, and *Nod*-Box NB15 Modulate Expression of the y4wEFG Locus That Is Required for Indole-3-Acetic Acid Synthesis in *Rhizobium* sp. strain NGR234. Mol Plant-Microbe Interact. 2004;17(10):1153–61.

11. Lorio JC, Chronis D, Krishnan HB. y4xP, an Open Reading Frame Located in a Type III Protein Secretion System Locus of *Sinorhizobium fredii* USDA257 and USDA191, Encodes Cysteine Synthase. Mol Plant-Microbe Interact. 2006;19(6):635–43.

12. D’Hooghe I, VanderWauven C, Michiels J, Tricot C, de Wilde P, Vanderleyden J, Stalon V. The Arginine Deiminase Pathway in *Rhizobium etli*: DNA Sequence Analysis and Functional Study of the *arcABC* Genes. J Bacteriol. 1997;179(23):7403–9.
