# Supplementary material for: Metabolic Analyses of Nitrogen Fixation in the Soybean Microsymbiont Sinorhizobium fredii Using Constraint-Based Modeling
Source: mSystems. 2020 Feb 18;5(1):e00516-19. doi: 10.1128/mSystems.00516-19 (PMC7029217; doi:10.1128/mSystems.00516-19)
Supplement: TABLE S9 [file mSystems.00516-19-st009.docx]

**Table S9**: List of references used to formulate the symbiosis reactions.

| **Compounds** | **References** |
| --- | --- |
| Carbon sources | (1,2) |
| Carbon storage | (1–4) |
| Fatty acids | (1,5,6) |
| Cofactors | (1,2,4,7–23) |
| Nitrogen fixation | (24,25) |
| Amino acids | (24–30) |

**References**

1. Li Y, Tian CF, Chen WF, Wang L, Sui XH, Chen WX. High-Resolution Transcriptomic Analyses of Sinorhizobium sp. NGR234 Bacteroids in Determinate Nodules of *Vigna unguiculata* and Indeterminate Nodules of *Leucaena leucocephala.* PLoS One. 2013;8(8).

2. Udvardi M, Poole PS. Transport and Metabolism in Legume-Rhizobia Symbioses. Annu Rev Plant Biol. 2013;64(1):781–805.

3. Poole P, Ramachandran V, Terpolilli J. Rhizobia: From saprophytes to endosymbionts. Nat Rev Microbiol. 2018;16(5):291–303.

4. Lodwig E, Poole P. Metabolism of Rhizobium bacteroids. CRC Crit Rev Plant Sci. 2003;22(1):37–78.

5. Brenner DJ, Krieg NR, Staley JT, editors. BERGEY’S MANUAL OF Systematic Bacteriology. Volume Two: The Proteobacteria. Second Edi. Springer; 2009.

6. Johnson G V, Evans HJ, Ching T. Enzymes of the glyoxylate cycle in rhizobia and nodules of legumes. Plant Physiol. 1966;41(8):1330–6.

7. Crespo-Rivas JC, Margaret I, Pérez-Montaño F, López-Baena FJ, Vinardell JM, Ollero FJ, Moreno FJ, Ruiz-Sainz JE, Buendía-Clavería AM. A *pyrF* auxotrophic mutant of *Sinorhizobium fredii* HH103 impaired in its symbiotic interactions with soybean and other legumes. Int Microbiol. 2007;10(3):169–76.

8. Wang D, Wang YC, Wu LJ, Liu JX, Zhang P, Jiao J, Yan H, Liu T, Tian CF, Chen WX. Construction and pilot screening of a signature-tagged mutant library of *Sinorhizobium fredii*. Arch Microbiol. 2016;198(2):91–9.

9. Jiang G, Krishnan AH, Kim Y, Wacek TJ, Krishnan HB, Acteriol JB. A Functional myo -Inositol Dehydrogenase Gene Is Required for Efficient Nitrogen Fixation and Competitiveness of *Sinorhizobium fredii* USDA191 To Nodulate Soybean ( *Glycine max* [ L .] Merr .). J Bacteriol. 2001;183(8):2595–604.

10. Jiao J, Ni M, Zhang B, Zhang Z, Young JPW, Chan F, Chen WX, Lam HM, Tian CF. Coordinated regulation of core and accessory genes in the multipartite genome of *Sinorhizobium fredii*. PLOS Genet. 2018;14(5):e1007428.

11. Medina C, Crespo-Rivas JC, Moreno J, Espuny MR, Cubo MT. Mutation in the *cobO* gene generates auxotrophy for cobalamin and methionine and impairs the symbiotic properties of *Sinorhizobium fredii* HH103 with soybean and other legumes. Arch Microbiol. 2009;191(1):11–21.

12. Oldroyd GED, Murray JD, Poole PS, Downie JA. The Rules of Engagement in the Legume-Rhizobial Symbiosis. Annu Rev Genet. 2011;45(1):119–44.

13. Kaiser BN, Moreau S, Castelli J, Thomson R, Lambert A, Bogliolo S, Puppo A, Day DA. The soybean NRAMP homologue, GmDMT1, is a symbiotic divalent metal transporter capable of ferrous iron transport. Plant J. 2003;35(3):295–304.

14. Dalton DA, Post CJ, Langeberg L. Effects of ambient oxygen and of fixed nitrogen on concentrations of glutathione, ascrobate, and associated enzymes in soybean root nodules. Plant Physiol. 1991;96(3):812–8.

15. Matamoros M a, Moran JF, Iturbe-Ormaetxe I, Rubio MC, Becana M. Glutathione and homoglutathione synthesis in legume root nodules. Plant Physiol. 1999;121(3):879–88.

16. El Msehli S, Lambert A, Baldacci-Cresp F, Hopkins J, Boncompagni E, Aschi Smiti S, Puppo A, Day DA. Crucial role of (homo) glutathione in nitrogen fixation in *Medicago truncatula* nodules. New Phytol. 2011;191:496–506.

17. Jiao J, Wu LJ, Zhang B, Hu Y, Li Y, Zhang XX, Guo HJ, Liu LX, Chen WX, Zhang Z, Tian CF. MucR is required for transcriptional activation of conserved ion transporters to support nitrogen fixation of *Sinorhizobium fredii* in soybean nodules. Mol Plant-Microbe Interact. 2016;29(5):352–61.

18. Clarke VC, Loughlin PC, Day DA, Smith PMC. Transport processes of the legume symbiosome membrane. Front Plant Sci. 2014;5:1–9.

19. Clarke VC, Loughlin PC, Gavrin A, Chen C, Brear EM, Day DA, Smith PM. Proteomic Analysis of the Soybean Symbiosome Identifies New Symbiotic Proteins. Mol Cell Proteomics. 2015;14(5):1301–22.

20. Randhawa GS, Hassani R. Role of rhizobial biosynthetic pathways of amino acids, nucleotide bases and vitamins in symbiosis. Indian J Exp Biol. 2002;40(7):755–64.

21. Kim CH, Kuykendall LD, Shah KS, Keister DL. Induction of Symbiotically Defective Auxotrophic Mutants of *Rhizobium fredii* HH303 by Transposon Mutagenesis. Appl Env Microbiol. 1988;54(2):423–7.

22. Buendía-Clavería AM, Moussaid A, Ollero FJ, Vinardell JM, Torres A, Moreno J, Gil-Serrano AM, Rodríguez-Carvajal MA, Tejero-Mateo P, Peart JL, Brewin NJ, Ruiz-Sainz JE. A *purL* mutant of *Sinorhizobium fredii* HH103 is symbiotically defective and altered in its lipopolysaccharide. Microbiology. 2003;149(7):1807–18.

23. Newman JD, Diebold RJ, Schultz BW, Noel KD. Infection of soybean and pea nodules by Rhizobium spp. purine auxotrophs in the presence of 5-aminoimidazole-4-carboxamide riboside. J Bacteriol. 1994;176(11):3286–94.

24. Li Y, Parsons R, Day DA, Bergersen FJ. Reassessment of major products of N_2_ fixation by bacteroids from soybean root nodules. Microbiology. 2002;148(6):1959–66.

25. Day DA, Poole PS, Tyerman SD, Rosendahl L. Ammonia and amino acid transport across symbiotic membranes in nitrogen-fixing legume nodules. Cell Mol Life Sci. 2001;58(1):61–71.

26. Fujihara S, Abe H, Minakawa Y, Akao S, Yoneyama T. Polyamines in nodules from various plant-microbe symbiotic associations. Plant Cell Physiol. 1994;35(8):1127–34.

27. Whitehead LF, Tyerman SD, Day DA. Polyamines as potential regulators of nutrient exchange across the peribacteroid membrane in soybean root nodules. Aust J Plant Physiol. 2001;28(7):677–83.

28. Dunn MF. Key roles of microsymbiont amino acid metabolism in rhizobia-legume interactions. Crit Rev Microbiol. 2014;41(4):411–51.

29. Kouchi H, Fukai K, Kihara A. Metabolism of glutamate and aspartate in bacteroids isolated from soybean root nodules. J Gen Microbiol. 1991;137(3):2901–10.

30. Waters JK, Hughes BL, Purcell LC, Gerhardt KO, Mawhinney TP, Emerich DW. Alanine, not ammonia, is excreted from N_2_-fixing soybean nodule bacteroids. Proc Natl Acad Sci. 1998;95(20):12038–42.
